# Impact of Dietary Enrichment with Omega-3 Polyunsaturated Fatty Acids from Extruded Linseed and *Padina pavonica* Algae Extract on Growth Performance and Metabolic Status in Fattening Rabbits

**DOI:** 10.3390/ani15142085

**Published:** 2025-07-15

**Authors:** Alda Quattrone, Doriana Beqiraj, Nour Elhouda Fehri, Rafik Belabbas, Daniele Vigo, Laura Menchetti, Olimpia Barbato, Sebastiana Failla, Massimo Faustini, Shereen Salama Ghoneim, Bayrem Jemmali, Simona Mattioli, Michela Contò, Albana Munga, Alessandro Dal Bosco, Imène Ben Salem, Enkeleda Ozuni, Mehmet Erman Or, Egon Andoni, Fabio Gualazzi, Marta Castrica, Gabriele Brecchia, Giulio Curone

**Affiliations:** 1 Department of Veterinary Medicine and Animal Sciences, University of Milan, Via dell’Università 6, 26900 Lodi, Italy; alda.quattrone@unimi.it (A.Q.); nour.fehri@unimi.it (N.E.F.); daniele.vigo@unimi.it (D.V.); massimo.faustini@unimi.it (M.F.); gabriele.brecchia@unimi.it (G.B.); giulio.curone@unimi.it (G.C.); 2 Faculty of Veterinary Medicine, Agricultural University of Tirana, Kodër Kamëz, 1029 Tirana, Albania; dkalamishi@yahoo.com (D.B.); amunga@ubt.edu.al (A.M.); enkelejda.ozuni@ubt.edu.al (E.O.); eandoni@ubt.edu.al (E.A.); 3 Laboratory of Research “Health and Animal Productions”, Higher National Veterinary School, Road Issad 26 Abes, Oued Smar, Algiers 16200, Algeria; r.belabbas@ensv.dz; 4 School of Biosciences and Veterinary Medicine, University of Camerino, Via Circonvallazione 93/95, 62024 Matelica, Italy; laura.menchetti@unicam.it (L.M.); fabio.gualazzi@studenti.unicam.it (F.G.); 5 Department of Veterinary Medicine, University of Perugia, Via San Costanzo 4, 06126 Perugia, Italy; 6 Consiglio per la Ricerca in Agricoltura e l’Analisi Dell’Economia Agraria (CREA), Research Centre for Animal Production and Aquaculture, Via Salaria 31, 00015 Rome, Italy; sebastiana.failla@crea.gov.it (S.F.); michela.conto@crea.gov.it (M.C.); 7 Animal Production Research Institute (APRI), Agriculture Research Center (ARC), Dokki, Giza 12651, Egypt; shereenghoneim25@yahoo.com; 8 LR13AGR02, Higher School of Agriculture, University of Carthage, Mateur 7030, Tunisia; jemmali.bayrem@gmail.com; 9 Department of Agricultural, Food and Environmental Science, University of Perugia, Borgo XX Giugno 74, 06124 Perugia, Italy; simona.mattioli@unipg.it (S.M.); alessandro.dalbosco@unipg.it (A.D.B.); 10 Département des Productions Animales, Service de Zootechnie et Economie Agricole Ecole Nationale de Médecine Vétérinaire, Université de la Manouba, Sidi Thabet 2020, Tunisia; bensalemimen@yahoo.fr; 11 Department of Internal Medicine, Faculty of Veterinary Medicine, Istanbul University-Cerrahpasa, Istanbul 34320, Türkiye; ermanor@iuc.edu.tr; 12 Department of Comparative Biomedicine and Food Science, University of Padova, Agripolis, Viale dell’Università 16, 35020 Legnaro, Italy; marta.castrica@unipd.it

**Keywords:** omega 3, PUFA, linseed, *Padina pavonica*, marine algae, metabolic hormones, growth performance, endocrine response

## Abstract

This study evaluated the effects of dietary supplementation with omega-3 polyunsaturated fatty acids from extruded linseed, alone and combined with *Padina pavonica* algae extract, on growth performance and metabolic status in fattening rabbits. Sixty New Zealand White rabbits were assigned to three groups, as follows: control (CNT), L (5% linseed), and LPP (3.5% linseed + 0.2% algae extract) from weaning (37 days) to slaughter (85 days). Productive performance was assessed through body weight (BW), average daily gain (ADG), feed conversion ratio (FCR), and feed intake (FI). Blood was sampled at weaning, 60 days, and slaughter and analyzed for insulin, leptin, cortisol, thyroid hormones (T3, T4), glucose, and non-esterified fatty acids (NEFAs). The L group showed significantly higher ADG (41.0 ± 1 g/d) and improved FCR (4.1 ± 0.2) compared to LPP (ADG: 37 ± 1 g/d, FCR: 4.6 ± 0.2; *p* = 0.001). No differences were observed in final BW or FI among groups (*p* < 0.001). Insulin peaked at 60 days across all groups (*p* < 0.001), with the LPP group showing the lowest levels (9.8 ± 0.9 µUI/mL; *p* = 0.043). T3 and T4 increased significantly with age (*p* < 0.001), and the T3/T4 ratio varied by diet and time (*p* = 0.005). Cortisol rose only at slaughter (*p* < 0.001) and negatively correlated with insulin and thyroid hormones. The results suggest that omega-3-rich nutraceuticals can enhance growth performance without disrupting metabolic balance and may modulate specific hormonal responses due to their bioactive compounds.

## Simple Summary

Omega-3 fatty acids are essential nutrients with significant nutraceutical properties that contribute to improved growth performance, reproductive efficiency, immune function, and meat quality in rabbits. Incorporating omega-3-rich sources, such as extruded linseed and marine algae like *Padina pavonica*, into rabbit diets has been shown to enhance the fatty acid profile of rabbit meat by increasing the polyunsaturated fatty acid (PUFA) content and optimizing the n-6/n-3 ratio. While numerous studies have demonstrated the benefits of various omega-3 sources on rabbit growth and meat quality, limited research has focused on their impact on metabolic status and related hormones and metabolites. Our study investigates the effects of these nutraceuticals, particularly *Padina pavonica*, which is rarely studied in animal nutrition, on rabbit growth performance and metabolic hormones, such as leptin, insulin, cortisol, T3, T4, glucose, and non-esterified fatty acids (NEFAs). By addressing this gap, our study seeks to provide a deeper understanding of how omega-3-rich diets, supplemented with extruded linseed and *Padina pavonica*, influence growth, metabolic processes, and overall health in rabbits, potentially improving both growth performance and meat quality.

## 1. Introduction

Enhancing the growth performance of fattening rabbits is essential for improving the economic sustainability of rabbit farming, particularly in light of the high costs associated with feed, which represent a major component of production expenses [1]. Productive performance in rabbits is influenced by several factors, including growth rate, health status, and metabolic efficiency [1]. Among the critical stages of rabbit production, weaning is particularly challenging, as it is associated with an increased risk of gastrointestinal disturbances [2]. These disorders can compromise nutrient absorption, slow growth, and increase the need for medical treatment, ultimately impairing animal health and farm profitability [3,4]. To support optimal growth and health during this vulnerable phase, it is crucial to provide a well-balanced diet that meets the energy and nutrient requirements of weanling rabbits, while preventing deficiencies and supporting immune function [2]. In this context, incorporating nutraceuticals as dietary supplements offers an innovative strategy to improve rabbit productivity and overall health [5]. Among these compounds, omega-3 polyunsaturated fatty acids (n-3 PUFAs) have received considerable attention for their broad range of physiological effects [1,6,7]. These essential fatty acids play a vital role in maintaining immune homeostasis by modulating inflammatory responses, improving gut health, and supporting physiological functions [1,8]. Furthermore, dietary supplementation with n-3 PUFAs has been shown to improve the nutritional profile of rabbit meat, potentially enhancing its value as a functional food with health benefits for human consumers [7,9].

In rabbit nutrition, the most common sources of omega-3 fatty acids include linseed, fish oil, and marine algae, each providing different types of essential fatty acids [7,10,11,12,13,14,15]. Linseed is particularly rich in alpha-linolenic acid (ALA, C18:3 n-3), which serves as a precursor for the synthesis of long-chain polyunsaturated fatty acids (PUFAs) such as eicosapentaenoic acid (EPA, C20:5 n-3), docosapentaenoic acid (DPA, 22:5 n-3), and docosahexaenoic acid (DHA, C22:6 n-3), although the conversion efficiency in mammals is limited [16]. Fish oil and marine algae, on the other hand, are direct sources of EPA and DHA, which are more bioavailable and play crucial roles in regulating inflammation, supporting immune function, and promoting overall health [8,17]. While most existing studies have documented the positive effects of these omega-3 sources on rabbit growth and meat quality [7,9,11,18,19], there is a lack of research focusing on their impact on metabolic status and related hormones and metabolites. In particular, although linseed has been widely used and studied in rabbit nutrition [10,12], its specific effects on the metabolism of growing rabbits remain inadequately explored. Incorporating linseed into rabbit diets has been shown to significantly modulate lipid metabolism, primarily by altering tissue fatty acid profiles [1,7,20]. Notably, linseed supplementation increases the content of n-3 PUFAs, particularly ALA, in muscle and adipose tissues, while concurrently reducing the n-6/n-3 PUFA ratio, a shift associated with improved health outcomes [7,19]. For instance, diets enriched with extruded linseed have demonstrated a decrease in saturated fatty acids and an increase in n-3 PUFAs in rabbit meat, enhancing its nutritional value [7,9]. Beyond tissue composition, linseed supplementation influences systemic lipid metabolism. Studies have reported that linseed-enriched diets lead to favorable changes in blood lipid profiles, including reductions in total cholesterol and low-density lipoprotein (LDL) cholesterol levels, which are beneficial for cardiovascular health [21,22,23]. Additionally, linseed oil supplementation has been associated with decreased triglyceride levels and improved high-density lipoprotein (HDL) concentrations in rabbits [21,24]. These metabolic effects underscore the potential of linseed as a valuable dietary component in rabbit nutrition, promoting enhanced growth performance, better health status, and superior meat quality in rabbits [1].

While linseed remains a well-established plant-based source of n-3 PUFAs in rabbit nutrition, recent research has expanded toward marine-derived alternatives, with particular interest in algae for their unique bioactive profiles and environmental sustainability [14,25]. Among these, *Padina pavonica*, a brown marine alga, has attracted scientific attention due to its rich composition of bioactive compounds and potential health benefits [6,7]. Notably, in addition to its nutritional value, *Padina pavonica* has demonstrated significant antidiabetic properties [26]. Studies indicate that its terpenoid-rich extracts can enhance insulin secretion and sensitivity, leading to improved glucose metabolism in diabetic animal models [26,27]. For instance, treatment with *Padina pavonica* extract ameliorated hyperglycemia and insulin resistance in type 2 diabetic rats, suggesting its potential role in metabolic regulation [26,27,28,29]. Beyond its metabolic effects, *Padina pavonica* is rich in phenolic compounds, flavonoids, vitamins, and phytosterols, contributing to its antioxidant, anti-inflammatory, and antimicrobial activities [26,30]. These bioactive constituents support various physiological functions, including enhancing nutrient absorption and modulating lipid profiles [15,28]. Additionally, *Padina pavonica* has been recognized for its ability to improve phosphorus and calcium absorption [31], which may be particularly beneficial during critical growth phases in animals, such as weaning and fattening [2]. Despite these promising attributes, research on the application of *Padina pavonica* in rabbit nutrition remains limited, and further studies are warranted to explore its potential benefits in enhancing rabbit health and productivity.

Given the increasing interest in nutraceuticals and their potential benefits for animal health, our hypothesis is that the combined supplementation of extruded linseed and *Padina pavonica* extract can improve growth performance and enhance metabolic efficiency in fattening rabbits.

This study aims to investigate the effects of dietary supplementation with extruded linseed, administered alone and in combination with *Padina pavonica* algae extract, on the growth performance and metabolic status of fattening rabbits. This study specifically focuses on assessing metabolic hormones and metabolites, which are critical indicators of energy balance and overall health, such as insulin, leptin, thyroid hormones (T3 and T4), and cortisol, along with metabolites including glucose and non-esterified fatty acids (NEFAs).

## 2. Materials and Methods

### 2.1. Animals, Diets, and Experimental Design

The growing rabbits used in this study were selected from the litters born to New Zealand White rabbit does that had been previously enrolled in an experimental trial involving the same dietary treatments [6]. Rabbits were reared under standard farming conditions in two commercial rabbit farms (Azienda Agricola Brachino Patrizia, Bagnoregio, Viterbo, Italy, and Azienda Agricola Borga Faeta, Caprese Michelangelo, Arezzo, Italy), in compliance with European legislation, specifically Legislative Decree No. 146, which implements Directive 98/58/EC on the protection of animals kept for farming purposes. The experimental protocol received approval from the Ethical Committee of the Department of Veterinary Medicine of the University of Milano (OPBA_18_2021, approved on 2 April 2021).

A total of sixty New Zealand White rabbit kits of mixed sexes were weaned at 37 days of age and individually housed in conventional cages (L × W × H: 75 × 38 × 25 cm) until slaughter at 85 days of age. Environmental conditions were kept constant throughout the trial, with a temperature range of 18–21 °C, relative humidity at 60%, and a controlled photoperiod of 16 h light and 8 h dark.

Upon weaning, the rabbits were assigned to one of three dietary groups (*n* = 20 per group), each receiving the same pelleted diet previously administered to their mothers [6,7]. The control group received a standard basal diet formulated to meet the nutritional requirements of growing rabbits (CNT diet) [7]. The other two groups were fed modified diets. One diet contained 5% extruded linseed (L) and another contained 3.5% extruded linseed plus 0.2% *Padina pavonica* algae extract (LPP). The algae extract was obtained from a commercial supplier and incorporated directly into the feed (Dietagro SAS, 11 Rue Nicolas Copernic, 53200 Château-Gontier-sur-Mayenne, France). In both experimental diets, the supplements replaced small portions of the ingredients in the basal control diet (Table 1), as described in detail in previous publications [6,7]. All diets were isoenergetic and formulated to ensure similar proximate chemical compositions, in accordance with nutritional recommendations for fattening rabbits [32]. Rabbits were fed daily with amounts gradually increasing from 100 g/day to 160 g/day throughout the study period. Fresh water was always available.

The proximate chemical composition and fatty acid profile of the experimental diets are presented in Table 2 and Table 3. The proximate chemical analysis was conducted following AOAC methods [33], while the fiber fraction was determined according to the procedure described by Van Soest et al. [34]. The fatty acid profile was analyzed using fatty acid methyl esters (FAMEs), whereas amino acid composition was determined using the AccQ•Tag Fluor reagent kit (Waters, Milford, MA, USA), according to previously described procedures [6,7].

At 85 days of age, rabbits were slaughtered in an authorized facility, complying with Council Regulation (EC) No. 1099/2009 on the protection of animals at the time of killing.

### 2.2. Productive Performance

Individual body weight (BW) was recorded every 8 days using an electronic scale (Isolad–Vignoli–Forlì, Forlì, Italy), in the morning before feeding.

Average daily gain (ADG) was determined using Equation (1):

ADG (g/d) = (Final Body Weight (g) − Initial Body Weight (g))/Number of Days(1)

Individual feed intake (FI) was recorded daily by measuring the amount of feed offered and subtracting the feed refusals.

The feed conversion ratio (FCR) was calculated using Equation (2):

FCR = (Total Feed Intake (g)/Total Weight Gain (g))(2)

### 2.3. Blood Sampling and Measurements of Hormones and Metabolites

From each animal, blood samples were collected three times during the experimental trial: on the day of weaning (T0: 37 days), at 60 days of age (T1), and at slaughter (T2: 85 days of age). Samples were drawn from the marginal ear vein into EDTA tubes, then immediately centrifuged at 3000× *g* for 15 min. The plasma was subsequently stored at −20 °C until analysis for hormones and metabolites. Cortisol, insulin, leptin, thyroid hormones (T3: triiodothyronine and T4: thyroxine), non-esterified fatty acids (NEFA), and glucose were analyzed from each sample, following the methods previously described by Menchetti et al. [36]. Briefly, leptin concentrations were measured using a double-antibody RIA with a multispecies leptin kit (Linco Research Inc., St. Charles, MO, USA). Plasma insulin was quantified using a double-antibody/PEG technique with a porcine insulin RIA kit (Linco Research Inc.), using guinea pig anti-porcine insulin antiserum and recombinant human insulin as the labeled antigen and standard. Total T3 and T4 were measured by RIA according to the manufacturer’s instructions (Immunotech, Prague, Czech Republic), while cortisol concentrations were assessed using the CORT kit (Immunotech). NEFA and glucose concentrations were analyzed following García-García et al. [37] and Rommers et al. [38], respectively. NEFA levels were determined using a two-reaction enzymatic-based colorimetric assay (Wako Chemicals GmbH, Neuss, Germany), and glucose concentrations were measured using the glucose oxidase method with the Glucose Infinity kit (Sigma Diagnostic Inc., St. Louis, MO, USA).

### 2.4. Statistical Analysis

Diagnostic graphs and Kolmogorov–Smirnov tests were used to check assumptions and identify outliers. Log transformation was used for BW, while Log(x + 1) was used for ADG and FCR. Raw data are presented as marginal means and in the figures. Extreme outliers were eliminated.

The Kruskal–Wallis test was used to compare feed intake among diets at each time point. The generalized estimating equations procedures were used to analyze hormones, where time was included as a within-subject effect (6 levels for productive parameters and 3 levels for hormones, from weaning until slaughter), and an unstructured correlation structure was specified to account for the correlation between repeated measures. Normal distribution and the identity link function were used for productive parameters (after transformation) and T3 concentrations, while gamma distribution and log link were used to analyze cortisol, insulin, leptin, T4, NEFA, glucose concentrations, and the T3/T4 ratio. The models evaluated the main effects of time (6 levels for productive parameters, and 3 levels for hormones), group (3 levels: CTN, L, and LPP groups), and the interaction between group and time. The BW at weaning was included as a covariate to evaluate the BW changes during growth. Sidak’s adjustment was used for multiple comparisons. The correlations among hormones and metabolites were evaluated using Spearman’s coefficient (ρ), and it was considered poor if ρ< |0.3|, medium if |0.3| ≤ ρ < |0.5|, and large if ρ ≥ |0.5|.

Statistical analyses were performed with SPSS Statistics version 25 (IBM, SPSS Inc., Chicago, IL, USA). We defined *p* ≤ 0.05 as significant.

## 3. Results

### 3.1. Productive Parameters

The rabbits’ body weight (BW) was influenced by time and weight at weaning (Table 4). The marginal means were 1566 ± 42, 1644 ± 42, and 1593 ± 39 g for CTN, L, and LPP, respectively, and multiple comparisons did not highlight specific differences.

No differences in feed intake (FI) were found between groups at any time point (for all time points: *p* > 0.1; Table 4).

Both the average daily gain (ADG) and feed conversion ratio (FCR) were influenced by group and time (Table 4). The marginal means of ADG for the L group (41.1 ± 1.0 g/d) were higher than those of LPP (37.0 ± 0.9 g/d; *p* = 0.002), even though multiple comparisons with Sidak’s correction did not reveal significant differences at individual time points. The highest peak of ADG was reached 5 weeks after weaning (46.6 ± 1.5 g/d). The LPP showed a higher marginal mean than L for FCR (4.0 ± 0.1, 3.9 ± 0.2, and 4.3 ± 0.1 for CTN, L, and LPP, respectively; *p* = 0.002), but multiple comparisons failed to detect differences at any time point.

### 3.2. Hormones and Metabolites

**Figure 1:**
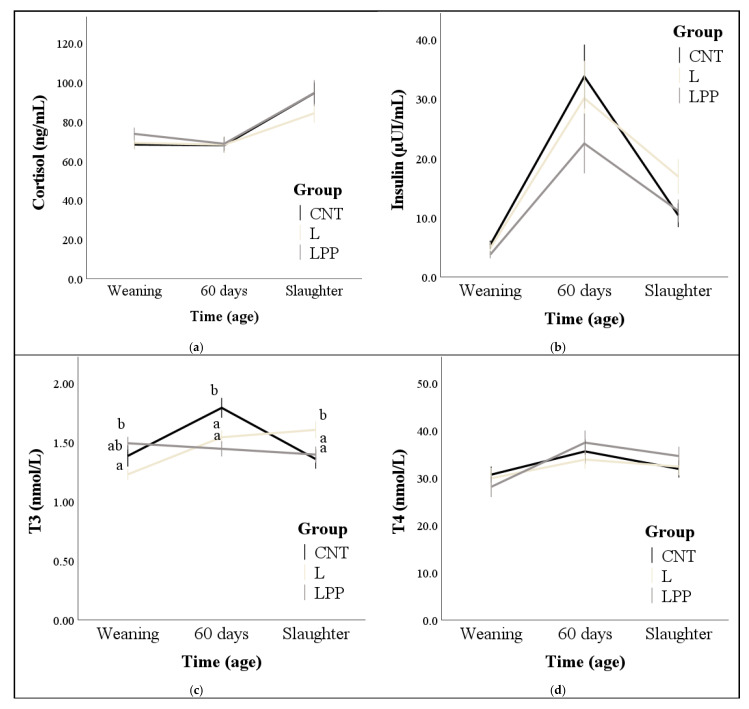

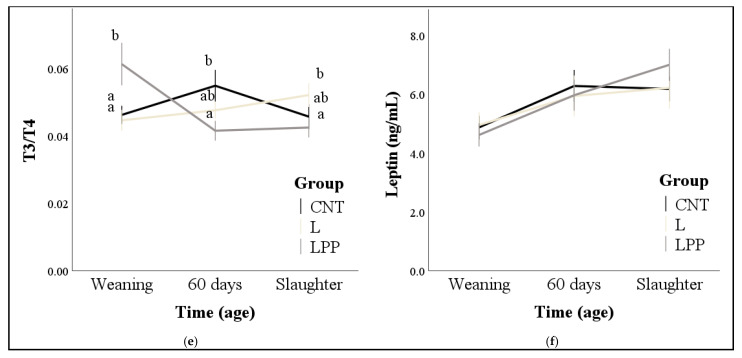
Effect of the standard diet (CNT), the standard diet integrated with 5% extruded linseed (L), and the standard diet integrated with 3.5% extruded linseed and 0.2% *Padina pavonica* algae extract (LPP) on cortisol, insulin, thyroid hormones, and leptin levels of fattening rabbits from weaning (37 days) until slaughter (85 days). Values are presented as means ± standard errors. Bars of standard errors that do not share the same letter at each time point differ significantly at *p* < 0.05. Panels without letters indicate that no significant differences were detected by multiple comparisons.

Regardless of group, an increase in cortisol concentrations was detected at slaughter compared to weaning (*p* < 0.001). Marginal means were 76.0 ± 2.3, 73.5 ± 2.6, and 78.2 ± 2.4 ng/mL for CTN, L, and LPP, respectively; however, the group and its interaction with time were not significant (Figure 1a). Moderate negative correlations were found at day 60 between cortisol and insulin, as well as between cortisol and thyroid hormones (*p* < 0.01; Table 5).

Regardless of group, insulin showed a peak at 60 days of age, while at slaughter, the concentrations were reduced but remained higher than at weaning (*p* < 0.001). Marginal means and group effect indicated that LPP had the lowest values for insulin concentrations (12.4 ± 1.0, 13.7 ± 1.5, and 9.8 ± 0.9 µUI/mL for CTN, L and LPP, respectively; *p* = 0.038), although the multiple comparisons did not highlight any differences, perhaps due to considerable variability in the data (Figure 1b). Moderate positive correlations were found between insulin and T3 at 60 days and slaughter, while it negatively correlated with cortisol at 60 days of age (*p* < 0.05; Table 5).

Regardless of the experimental group, both T3 (Figure 1c) and T4 (Figure 1d) concentrations increased significantly at 60 days compared to weaning (*p* < 0.01), with no significant differences observed between groups overall. However, T3 levels exhibited a distinct temporal pattern among groups (group × time effect: *p* < 0.001); values were higher in the LPP group at weaning (compared to the L group), and in the CNT and L groups at 60 days and at slaughter, respectively (compared to the other two groups; *p* < 0.05). The T4 concentrations were similar among the groups, while a significant interaction effect of group × time was found for the T3/T4 ratio (*p* = 0.005; Figure 1e). Specifically, at weaning, the LPP group exhibited the highest T3/T4 ratio (*p* < 0.05). At 60 days, the control group showed a higher ratio than the LPP group, while at slaughter, the L group presented a higher ratio compared to the LPP group (*p* < 0.05).

Leptin was influenced only by age, with marginal means increasing progressively from 4.8 ± 0.2 ng/mL at weaning to 6.5 ± 0.3 ng/mL at slaughter (*p* < 0.001) without differences between groups (Figure 1f).

**Figure 2:**
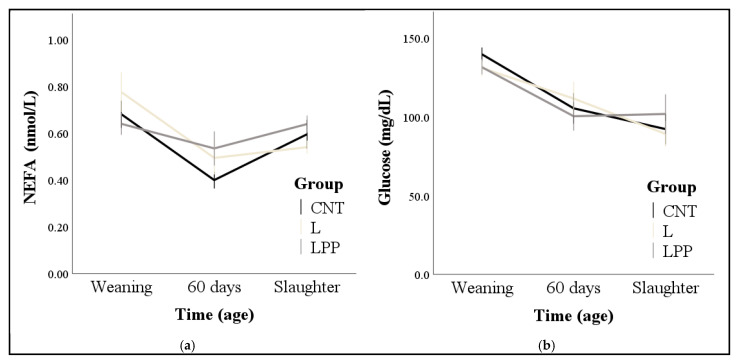
Effects of the standard diet (CNT), the standard diet integrated with 5% extruded linseed (L), and the standard diet integrated with 3.5% extruded linseed and 0.2% *Padina pavonica* algae extract (LPP) on NEFA (Panel **a**) and glucose (Panel **b**) levels of fattening rabbits from weaning (37 days) until slaughter (85 days). Values are means ± standard errors.

NEFA concentrations were influenced by time and, regardless of group, the lowest values were found at 60 days (0.47 ± 0.03 nmol/L; *p* < 0.001). NEFA concentrations were also influenced by group × time interaction (*p* = 0.013), but multiple comparisons did not highlight differences among groups. Marginal means were 0.55 ± 0.03, 0.59 ± 0.03, and 0.60 ± 0.03 nmol/L for CTN, L, and LPP, respectively (Figure 2a). NEFA concentrations correlated positively with leptin at weaning and 60 days (correlations ranged from medium to strong), while they correlated negatively with insulin at 60 days and at slaughter and with thyroid hormones at all the time points (correlations ranged from poor to medium; *p* < 0.05; Table 5).

Glucose levels were only affected by time, with the highest values observed at weaning (140.0 ± 2.7 mg/dL; *p* < 0.001). Neither the group (110.7 ± 6.1, 109.2 ± 6.4, and 110.3 ± 6.7 mg/dL for CTN, L, and LPP, respectively) nor its interaction with time was significant (Figure 2b). Glucose correlated positively with cortisol at weaning, with T4 at 60 days, and with cortisol and insulin at slaughter (*p* < 0.05; Table 5). Moderate negative correlations were found for glucose with leptin and NEFA at weaning and slaughter (*p* < 0.05; Table 5).

## 4. Discussion

Growth performance and metabolic regulation are intrinsically linked, as efficient nutrient utilization and energy balance are essential for optimal weight gain and feed conversion [1]. Proper metabolic function, mediated through hormonal and biochemical pathways, supports tissue growth, whereas dysregulation can impair performance and compromise animal health [36]. Therefore, in our study, we evaluated both productive performance and metabolic status to obtain a comprehensive understanding of the animals’ physiological responses to the different dietary treatments.

Previous studies have generally reported minimal effects of n-3 PUFAs on the growth performance of fattening rabbits, regardless of the source, such as linseed or marine algae [1,10,39]. Consistent with these findings, our study observed no significant differences in body weight among the experimental groups. However, the marginal means for both ADG and FCR were significantly affected by the dietary treatment, although no significant differences were observed at individual time points. Rabbits fed the diet supplemented with 5% extruded linseed exhibited higher ADG and improved FCR compared to those receiving the combined diet of 3.5% extruded linseed and 0.2% *Padina pavonica* extract. These results suggest that supplementing rabbit diets with 5% extruded linseed may enhance growth performance and nutrient utilization more effectively than when combined with *Padina pavonica*. One plausible explanation for this observation is the high ash content present in marine algae such as *Padina pavonica* [40], even though the inclusion level used in our study was low. Marine algae are indeed recognized for their high mineral content, including essential elements like calcium and phosphorus [14,15,41]. While these minerals are vital for various physiological functions, their excessive presence can dilute the energy density of the diet, potentially leading to reduced feed efficiency [42]. Moreover, the high ash content in marine algae may interfere with nutrient digestibility and absorption, thereby potentially reducing the availability of nutrients to the animal [43]. Additionally, it is worth noting that the linseed content in the LPP group (3.5%) was lower than in the L group (5%), which may also contribute to the observed differences in productive performance. The reduced inclusion level of linseed in the LPP group could have limited its potential benefits on growth and feed efficiency. Based on these findings, it may be suggested that the inclusion level of linseed should not fall below 5% in order to exert a consistent positive effect on performance. Despite these considerations, it is important to highlight that, in our study, the inclusion of *Padina pavonica* did not adversely affect the body weight or feed intake of the growing rabbits, indicating that the diet’s palatability remained acceptable [15,25]. The absence of significant differences in feed intake observed throughout the entire experimental trial is a key outcome in the context of rabbit production, as any reduction in feed intake can compromise body condition, leading to decreased weight gain and potentially affecting meat quality [32]. Previous studies have raised concerns that certain marine algae may reduce palatability, leading to decreased feed intake in rabbits [44,45], probably due to the fact that the high ash content and the presence of bioactive compounds may introduce off-flavors or odors into the feed [14,46]. Conversely, our results indicate that the addition of 0.2% *Padina pavonica* extract does not impair feed intake, suggesting that marine algae, when used at appropriate levels, can be effectively integrated into rabbit diets without affecting palatability. This valuable result supports the use of marine algae as sustainable and functional feed ingredients capable of delivering health benefits without detrimental effects on feed intake or overall animal performance.

To gain deeper insight into the physiological impact of the dietary treatments, we measured circulating levels of key metabolic hormones and metabolites that are crucial for growth, development, and overall productive performance in fattening rabbits. These biomarkers are integral to energy homeostasis and metabolic regulation [36]. Specifically, insulin and glucose levels reflect carbohydrate metabolism and energy availability, while leptin and NEFAs are indicative of lipid mobilization and adipose tissue status [36]. In addition, thyroid hormones are fundamental for regulating basal metabolic rate, and cortisol is a well-established marker of both stress response and energy metabolism [36]. In our study, plasma cortisol levels increased only at slaughter, consistent with the activation of the hypothalamic–pituitary–adrenal (HPA) axis in response to acute stress [47,48]. This elevation in cortisol is a common physiological response during the slaughter process, as observed in various animal species, including rabbits [47]. Throughout the entire experimental trial, plasma cortisol levels remained within physiological ranges in all groups, with no significant differences observed, suggesting similar stress responses [49]. At 60 days of age, a moderate negative correlation was observed between cortisol and both insulin and thyroid hormone levels. Specifically, lower cortisol concentrations were associated with higher levels of insulin and thyroid hormones. This finding is consistent with physiological mechanisms in healthy individuals, where insulin and cortisol act as major antagonistic regulators of energy homeostasis [50]: insulin suppresses appetite and consequently reduces food intake, while cortisol stimulates appetite [51]. The observed inverse relationship suggests that reduced stress levels at this growth stage may favor anabolic processes mediated by anabolic hormones such as insulin and thyroid hormones, which are essential for promoting growth and regulating metabolism [52]. In contrast, elevated cortisol levels can suppress insulin secretion and reduce peripheral sensitivity to insulin, impairing glucose uptake and utilization [53]. Similarly, chronic stress and elevated glucocorticoids can inhibit the hypothalamic–pituitary–thyroid axis, resulting in decreased thyroid hormone production [54]. Therefore, maintaining lower cortisol levels during this critical growth period may help preserve hormonal balance and enhance overall growth performance [55]. Moreover, monitoring key metabolic hormones such as insulin and T3, alongside cortisol, is essential for a comprehensive evaluation of animal welfare [56].

In this study, insulin concentrations exhibited a peak at 60 days of age across all groups, followed by a reduction at slaughter, when, however, these levels remained higher than those observed at weaning. This pattern suggests an age-related modulation of insulin secretion, potentially reflecting the animals’ metabolic adaptations during growth and development [57]. Such trends align with findings in other species, where insulin levels increased with age and body weight, indicating enhanced anabolic activity during growth phases [58]. We hypothesize that the lower insulin concentrations observed in the LPP group may result from the bioactive compounds present in this brown seaweed [59,60], which is known to influence glucose metabolism and insulin sensitivity [26]. *Padina pavonica* is rich in terpenoids that activate peroxisome proliferator-activated receptor gamma (PPARγ), a nuclear receptor involved in glucose and lipid metabolism [27]. Activation of PPARγ enhances insulin sensitivity, leading to improved glucose uptake by tissues and a consequent reduction in circulating insulin levels [27]. Additionally, extracts from *Padina pavonica* have demonstrated inhibitory effects on α-amylase and α-glucosidase, enzymes responsible for the breakdown of complex carbohydrates into glucose [29]. By inhibiting these enzymes, *Padina pavonica* slows carbohydrate digestion and glucose absorption, leading to a reduced postprandial blood glucose rise and decreased insulin secretion [29]. Furthermore, *Padina pavonica* is rich in soluble dietary fibers, which increase the viscosity of the intestinal contents, slowing nutrient absorption and attenuating the glycemic response [27,61], thereby reducing the need for elevated insulin secretion [27].

Additionally, insulin levels showed a moderate positive correlation with T3 concentrations at both 60 days and at slaughter. T3, the biologically active form of thyroid hormone, plays a central role in metabolic regulation and has been shown to improve insulin sensitivity [53] and promote glucose utilization [62]. This correlation, once again, highlights the functional interplay between thyroid activity and insulin-mediated metabolic control during growth. Both T3 and T4 concentrations significantly increased at 60 days of age across all experimental groups, reflecting normal physiological maturation in growing rabbits [36]. This rise aligns with the increased metabolic demands associated with growth, as thyroid hormones play a crucial role in regulating energy metabolism and tissue development [54]. While T4 levels remained consistent among the dietary groups, variations in T3 concentrations and the T3/T4 ratio were observed over time and between groups, suggesting that dietary components may influence peripheral conversion of T4 to the more active T3. Notably, at weaning, the group receiving both extruded linseed and *Padina pavonica* extract (LPP) exhibited higher T3 levels compared to the group receiving only linseed. We hypothesize that this difference may be attributed to the bioactive compounds present in *Padina pavonica*, known to contain iodine [15], which is a key element in thyroid hormone synthesis [63]. The significant interaction effect observed in the T3/T4 ratio indicates that the balance between T3 and T4 shifted over time and among groups. At weaning, the LPP group showed the highest T3/T4 ratio, indicating enhanced conversion of T4 to T3, potentially due to the synergistic effects of linseed-derived omega-3 fatty acids and seaweed bioactive compounds. However, at 60 days and at slaughter, the control and L groups, respectively, exhibited higher ratios compared to the LPP group, which may reflect adaptive metabolic responses to their specific diets or developmental stages.

Leptin concentrations exhibited a significant age-related increase from weaning to slaughter across all dietary groups. This progression aligns with the physiological growth and associated expansion of adipose tissue in fattening rabbits [64,65]. Importantly, no significant differences were observed among dietary groups, a result that aligns with the similar body weights recorded across all groups, indicating comparable adipose tissue accumulation [66]. Leptin, predominantly secreted by adipocytes, functions as a key regulator of energy homeostasis by signaling energy reserves to the hypothalamus, thereby modulating appetite and energy expenditure [67]. Circulating leptin levels are closely correlated with fat mass, increasing as adipose tissue accumulates [68]. Overall, the progressive increase in leptin levels with age highlights the hormone’s function in reflecting energy reserves and regulating metabolic processes associated with growth [36]. These results emphasize the importance of considering age-related physiological changes when evaluating hormonal responses to dietary interventions in growing animals.

Circulating NEFA levels, critical indicators of lipid mobilization and energy status [36,69], exhibited significant temporal variations, with the lowest concentrations observed at 60 days of age. The absence of significant differences in NEFA concentrations among groups suggests that diets integrated with linseed and algae do not impair energy metabolism, as evidenced by the similar levels of lipolysis compared to the control group. Notably, we found a strong positive correlation between NEFA and leptin concentrations at both weaning and 60 days, suggesting that adipocyte-derived leptin may rise in parallel with enhanced lipid mobilization during these key developmental stages [70,71]. Additionally, a moderate negative correlation was observed between NEFA and insulin concentrations. This finding aligns with one of the established roles of insulin as an anti-lipolytic hormone, suppressing the release of NEFA from the adipose tissue into circulation [72].

Finally, plasma glucose concentrations were significantly influenced by age, with the highest levels observed at weaning. This age-related peak is consistent with known developmental changes in insulin sensitivity and glucose metabolism in young animals, with immature pancreatic function and hormonal regulation [73]. Importantly, no significant differences in glucose concentrations were detected among the dietary groups throughout the study period. This finding suggests that supplementation with 5% extruded linseed and/or 0.2% *Padina pavonica* extract did not adversely affect glucose metabolism in healthy, growing rabbits. Such stability underscores the robustness of glucose homeostasis mechanisms in healthy individuals, where regulatory systems effectively maintain normoglycemia despite dietary variations.

## 5. Conclusions

Dietary supplementation with 5% extruded linseed improved growth performance in fattening rabbits, enhancing average daily gain and feed conversion efficiency without adversely affecting metabolic balance. The addition of *Padina pavonica* extract at 0.2% did not impair feed intake or body weight but modulated hormonal responses, particularly insulin. These findings support the safe inclusion of omega-3-rich nutraceuticals in rabbit diets, with consideration for the specific effects of combined supplements on metabolic parameters. Monitoring these parameters provides insights into how dietary interventions influence metabolic pathways, thereby affecting the growth performance and health status of fattening rabbits. Such assessments are essential for optimizing feeding strategies to enhance productivity and animal welfare. However, further studies are needed to explore the underlying mechanisms by which these supplements influence metabolic and hormonal responses. Such research will contribute to the development of evidence-based feeding strategies that promote sustainable and efficient rabbit production.

## Figures and Tables

**Table 1:** Formulation (%) of the experimental diets of the different experimental groups: standard diet (CNT), standard diet integrated with 5% extruded linseed (L), and standard diet integrated with 3.5% extruded linseed and 0.2% *Padina pavonica* algae extract (LPP).

Ingredients (%)	CNT	L	LPP
Extruded linseed	-	5	3.5
“*Padina Pavonica*” algae extract	-	-	0.2
Wheat bran	23.16	23.09	23.08
Beet pulp	11.5	9.33	11
Wheat straw	11	11	11
Alfalfa	10	12.5	10.17
Sunflower husks	9.95	6	10.78
Barley	9.5	9	9.5
Sunflower seed meal	8.83	14	9.17
Soybean hulls	0.17	-	-
Toasted soybean seed	5	-	1.50
Sugarcane molasses	3	3	3
Wheat	2.5	2.5	2.5
Grape seed meal	2.17	1.83	1.87
Soybean oil	0.55	-	-
Palm oil	0.33	0.33	0.33
Carboxymethylcellulose	0.2	0.2	0.2
Liquid acidifier ^1^	0.15	0.15	0.15
Calcium carbonate	0.8	0.8	0.8
Sodium chloride	0.4	0.4	0.4
Magnesium oxide	0.15	0.15	0.15
Oligo-vitamin supplement ^2^	0.25	0.25	0.25
Methionine hydroxy analog	0.15	0.14	0.15
Lysine	0.14	0.21	0.19
L Threonine	0.07	0.08	0.08
Vitamin E 50%	0.03	0.03	0.03

^1^ Liquid acidifier composition: formic acid 75%. ^2^ Oligo-vitamin supplement premix composition per kg of diet: vitamin A 11,000 IU; vitamin D3 2000 IU; vitamin B1 2.5 mg; vitamin B2 4 mg; vitamin B6 1.25 mg; vitamin B12 0.01 mg; alpha-tocopherol acetate 50 mg; biotin 0.06 mg; vitamin K 2.5 mg; niacin 15 mg; folic acid 0.30 mg; D-pantothenic acid 10 mg; choline 600 mg; Mn 60 mg; Fe 50 mg; Zn 15 mg; I 0.5 mg; Co 0.5 mg.

**Table 2:** Chemical composition (g/100 g) of the experimental diets: standard diet (CNT), standard diet integrated with 5% extruded linseed (L), and a standard diet integrated with 3.5% extruded linseed combined with 0.2% *Padina pavonica* algae extract (LPP).

Chemical Composition (g/100 g)	CNT	L	LPP
Dry matter	88.61	88.89	88.85
Crude protein	15.03	15.28	15.13
Crude fat	3.56	3.75	3.57
Ash	7.95	8.04	8.10
Crude fiber	18.64	18.63	18.71
NDF ^1^	36.13	36.22	36.15
ADF ^2^	22.57	22.62	22.57
ADL ^3^	6.50	6.50	6.50
Met + Cys ^4^	0.6	0.6	0.6
Lys ^5^	0.7	0.7	0.7
Thr ^6^	0.58	0.58	0.58
Digestible energy ^7^	2189.1	2190.4	2188.7

^1^ NDF: neutral detergent fiber; ^2^ ADF: acid detergent fiber; ^3^ ADL: acid detergent lignin; ^4^ Met + Cys: Methionine + cysteine; ^5^ Lys: lysine; ^6^ Thr: threonine. ^7^ as Kcal/kg. Estimated by Maertens et al. [35].

**Table 3:** Fatty acids profile (% of total fatty acids) of the experimental diets: standard diet (CNT), standard diet integrated with 5% extruded linseed (L), and standard diet integrated with 3.5% extruded linseed and 0.2% *Padina pavonica* algae extract (LPP).

	CNT	L	LPP
14:0	0.33	0.29	0.32
16:0	16.75	15.74	16.11
16:1 n-7	0.19	0.23	0.21
17:0	0.12	0.11	0.09
18:0	6.80	6.94	7.18
18:1 n-9	19.41	18.63	18.60
18:1 n-7	1.25	1.01	1.20
18:2 n-6, LA ^1^	47.25	33.32	33.54
20:0	0.32	0.29	0.26
18:3 n-6, γ-ALA ^2^	0.23	0.30	0.25
18:3 n-3, α-ALA	5.35	21.71	20.42
22:1 n-11	0.17	0.17	0.17
20:4 n-6, AA ^3^	0.06	0.05	0.19
20:5 n-3, EPA ^4^	-	-	0.09
22:5 n-3 DPA ^5^	-	-	0.06
SFA ^6^	25.26	24.21	24.85
MUFA ^7^	21.79	20.36	20.54
PUFA n-6 ^8^	47.60	33.72	34.04
PUFA n-3 ^9^	5.35	21.71	20.58

^1^ LA = linoleic acid; ^2^ ALA = linolenic acid; ^3^ AA = arachidonic acid, ^4^ EPA = eicosapentaenoic acid; ^5^ DPA = docosapentaenoic acid; ^6^ SFA = saturated fatty acids ∑14:0, 16:0, 17:0, 18:0, 20:0; ^7^ MUFA = monounsaturated fatty acids ∑16:1, 18:1n-9, 18:1 n-7, 22:1 n-11; ^8^ PUFA n-6 = polyunsaturated fatty acid omega 6 ∑18:2 n-6, 18:3 n-6, 20:4 n-6; ^9^ PUFA n-3 = polyunsaturated fatty acid omega 3 ∑18:3 n-3, 20:5 n-3, 22:5 n-3.

**Table 4:** Effect of the standard diet (CNT), the standard diet integrated with 5% extruded linseed (L), and the standard diet integrated with 3.5% extruded linseed and 0.2% *Padina pavonica* algae extract (LPP) on the productive parameters of fattening rabbits from weaning (37 days of age, week 0) until slaughter (85 days of age, week 6 post-weaning). Values are means ± standard errors. For each parameter and each time point, means that do not share the same letter are significantly different at *p* < 0.05.

Parameter	Group	Week Post-Weaning							Significance			
		0	1	2	3	4	5	6	BW at Weaning	Group	Time	Group × Time
BW	CNT	840 ± 26	1088 a ± 31	1315 a ± 31	1551 a ± 40	1828 a ± 47	2128a ± 61	2445 a ± 64	<0.001	0.397	<0.001	0.588
	L	863 ± 23	1122 a ± 22	1362 a ± 20	1633 a ± 20	1888 a ± 34	2223a ± 33	2529 a ± 29				
	LPP	889 ± 32	1126 a ± 40	1344 a ± 38	1560 a ± 38	1816 a ± 47	2151a ± 47	2436 a ± 53				
Feed intake (g)	CNT	100 ± 0	110 ± 0	120 ± 0	135 ± 0	150 ± 0	160 ± 0	160 ± 0	- *	- *	- *	- *
	L	100 ± 0	110 ± 0	120 ± 0	135 ± 0	150 ± 0	160 ± 0	160 ± 0				
	LPP	100 ± 0	110 ± 0	120 ± 0	135 ± 0	150 ± 0	160 ± 0	160 ± 0				
ADG (g/d)	CNT	-	36.6 a ± 2.25	32.5 a ± 2.1	33.7 a ± 2.0	40.6 a ± 2.0	47.3 a ± 2.8	41.6 a ± 4.3	-	0.002	<0.001	0.958
	L	-	38.4 a ± 2.06	34.3 a ± 2.5	38.6 a ± 2.2	40.7 a ± 1.7	49.3 a ± 2.1	45.6 a ± 4.4				
	LPP	-	33.8 a ± 2.32	31.3 a ± 2.2	30.8 a ± 1.9	42.8 a ± 2.6	43.4 a ± 3.1	41.3 a ± 3.2				
FCR	CNT	-	3.51 a ± 0.41	4.15 a ± 0.30	4.47 a ± 0.35	3.63 a ± 0.32	3.63 a ± 0.32	4.91 a ± 0.58	-	0.003	<0.001	0.695
	L	-	4.18 a ± 1.08	4.22 a ± 0.43	3.94 a ± 0.39	3.80 a ± 0.22	3.80 a ± 0.22	4.06 a ± 0.29				
	LPP	-	3.68 a ± 0.23	4.63 a ± 0.43	5.00 a ± 0.38	4.09 a ± 0.36	4.09 a ± 0.36	4.50 a ± 0.39				

* This parameter was analyzed using the Kruskal–Wallis test at each time point, and no significant differences were found.

**Table 5:** Correlation coefficients (Spearman’s rho) between hormones and metabolites for each time point.

Time (Age)		Cortisol	Insulin	T3	T4	Leptin	NEFA
Weaning	Insulin	−0.028	--				
	T3	−0.037	−0.101	--			
	T4	0.105	0.216	0.168	--		
	Leptin	0.139	0.208	0.063	−0.160	--	
	NEFA	0.231	−0.062	−0.214	**−0.256** *	**0.465** **	--
	Glucose	**0.332** **	0.113	−0.101	0.017	0.024	**0.377** **
60 days of age	Insulin	**−0.450** **	--				
	T3	**−0.364** **	**0.369** **	--			
	T4	**−0.405** **	0.252	**0.421** **	--		
	Leptin	0.106	0.019	−0.031	−0.090	--	
	NEFA	0.217	−0.152	**−0.298** *	**−0.427** **	**0.512** **	--
	Glucose	−0.169	0.042	0.063	**0.286** *	**−0.484** **	**−0.574** **
Slaughter	Insulin	0.233	--				
	T3	0.136	**0.330** *	--			
	T4	−0.067	0.212	0.175	--		
	Leptin	−0.235	−0.121	−0.032	0.049	--	
	NEFA	−0.061	**−0.280** *	**−0.292** *	0.012	0.239	--
	Glucose	**0.389** **	**0.259** *	0.194	−0.196	−0.209	**−0.335** **

The coefficients significant at *p* < 0.05 are indicated in bold; *. Correlation is significant at the 0.05 level (2-tailed). **. Correlation is significant at the 0.01 level (2-tailed).

## Data Availability

The original contributions presented in this study are included in the article.
